# Improved detection of molecularly targeted iron oxide particles in mouse brain using *B*_0_ field stabilised high resolution MRI

**DOI:** 10.1016/j.mri.2020.01.002

**Published:** 2020-04

**Authors:** Stuart Gilchrist, Paul Kinchesh, Niloufar Zarghami, Alexandre A. Khrapitchev, Nicola R. Sibson, Veerle Kersemans, Sean C. Smart

**Affiliations:** Cancer Research UK and Medical Research Council Oxford Institute for Radiation Oncology, Department of Oncology, University of Oxford, United Kingdom

**Keywords:** *B*_0_ correction, *B*_0_ lock, High resolution, Molecular imaging, Iron oxide particles

## Abstract

**Purpose:**

High resolution multi-gradient echo (MGE) scanning is typically used for detection of molecularly targeted iron oxide particles. The images of individual echoes are often combined to generate a composite image with improved SNR from the early echoes and boosted contrast from later echoes. In 3D implementations prolonged scanning at high gradient duty cycles induces a *B*_0_ shift that predominantly affects image alignment in the slow phase encoding dimension of 3D MGE images. The effect corrupts the composite echo image and limits the image resolution that is realised. A real-time adaptive *B*_0_ stabilisation during respiration gated 3D MGE scanning is shown to reduce image misalignment and improve detection of molecularly targeted iron oxide particles in composite images of the mouse brain.

**Methods:**

An optional *B*_0_ measurement block consisting of a 16 μs hard pulse with FA 1°, an acquisition delay of 3.2 ms, followed by gradient spoiling in all three axes was added to a respiration gated 3D MGE scan. During the acquisition delay of each *B*_0_ measurement block the NMR signal was routed to a custom built *B*_0_ stabilisation unit which mixed the signal to an audio frequency nominally centred around 1000 Hz to enable an Arduino based single channel receiver to measure frequency shifts. The frequency shift was used to effect correction to the main magnetic field via the *B*_0_ coil. The efficacy of *B*_0_ stabilisation and respiration gating was validated in vivo and used to improve detection of molecularly targeted microparticles of iron oxide (MPIO) in a mouse model of acute neuroinflammation.

**Results:**

Without *B*_0_ stabilisation 3D MGE image data exhibit varying mixtures of translation, scaling and blurring, which compromise the fidelity of the composite image. The real-time adaptive *B*_0_ stabilisation minimises corruption of the composite image as the images from the different echoes are properly aligned. The improved detection of molecularly targeted MPIO easily compensates for the scan time penalty of 14% incurred by the *B*_0_ stabilisation method employed. Respiration gating of the *B*_0_ measurement and the MRI scan was required to preserve high resolution detail, especially towards the back of the brain.

**Conclusions:**

High resolution imaging for the detection of molecularly targeted iron oxide particles in the mouse brain requires good stabilisation of the main *B*_0_ field, and can benefit from a respiration gated image acquisition strategy.

## 1 Introduction

Molecular MRI is an evolving field of imaging with strong translational and research potential. The ability to detect neuroinflammatory biomarkers in vivo allows cellular events related to disease processes to be studied and may be used to inform decisions about treatments and to monitor responses to treatments. Microparticles of iron oxide (MPIO) targeting adhesion molecules that are expressed on endothelial cells are the preferred contrast agents for imaging neuroinflammation in preclinical models [[1], [2], [3]]. It has been shown that vascular cell adhesion molecule (VCAM-1) is a robust target for many disease models such as acute inflammation, Alzheimer's disease, cerebral ischemia and secondary brain cancer [[4], [5], [6], [7], [8], [9]].

Targeted molecular MRI combines the advantages of high spatial resolution and contrast without the need for ionising radiation making it an attractive imaging technique to study molecular processes. Individual iron oxide particles have been observed to generate a field distortion over a region that is at least 50 times the size of the particle [10], which makes the close proximity of only a few MPIO detectable by high resolution *T*_2_*-weighted MRI. High resolution multi-gradient echo (MGE) scanning is typically used for efficient MPIO detection, and the echoes are often combined to generate a composite image with improved signal-to-noise ratio (SNR) from the early echoes and boosted contrast from later echoes, as in the MEDIC/MERGE/M-FFE/ADAGE methods [[11], [12], [13]].

3D implementations enable images to be generated with isotropic resolution albeit at the expense of longer scan times. However, prolonged scanning at high gradient duty cycles can heat ferromagnetic components within the magnet system which modifies the magnetic permeability and induces a time-dependent, non-linear *B*_0_ shift. For 3D MGE imaging this predominantly affects image alignment in the slow phase encode direction [14]. The problem can be particularly severe if a significant amount of passive shimming is used to achieve base level homogeneity of the main magnetic field. The effect corrupts the composite echo image and limits the image resolution that is realised, which compromises the detection and quantitation of MPIO-induced effects. Previous studies in our laboratory have largely been performed on a system where the *B*_0_ drift is known to be relatively mild [14]. Nevertheless, good care was always taken to pre-warm the system prior to data acquisition to enable accurate detection of MPIO-induced effects in the composite echo images, which incurred a 50–100% time penalty [3,9].

The importance of field-frequency locking superconducting magnets during high-resolution NMR has long been appreciated [15] and quickly became the default mode of operation. In MRI the requirement is not so stringent, but its benefit is becoming increasingly apparent for EPI, RARE, balanced SSFP, phase contrast and spiral scan modes on clinical scanners [[16], [17], [18], [19], [20]]. Such corrections are not incorporated in any of the supplied MRI scan modes on preclinical systems. This paper demonstrates that a real-time adaptive *B*_0_ stabilisation in conjunction with respiratory gated 3D MGE scanning reduces the effect of image misalignment and improves high resolution detection and visualisation of VCAM-MPIO in a mouse model of acute neuroinflammation. The method of *B*_0_ stabilisation deployed effects adjustment to a *B*_0_ coil in a similar manner to that described previously on a whole body system [21] except that the absence of a mechanism to enable real time processing of reference scan data acquired internally by the spectrometer necessitated development of an external hardware solution.

## 2 Methods

### 2.1 MRI

MRI was performed on a 9.4 T 160 mm horizontal bore VNMRS preclinical imaging system equipped with 100 mm bore gradient insert (Varian Inc., CA). RF transmission and reception was performed with a 30 mm long 25 mm ID quadrature birdcage coil (Rapid Biomedical GmbH, Germany).

Respiration gating with in-line reacquisition of respiration corrupted data was implemented as an option in a steady-state MGE 3D scan, in a fashion related to that described previously [21]. Gating signal evaluation was performed following each multi-echo block which consisted of a 16 μs hard pulse FA 12° and acquisition of 8 gradient echoes all in the same k-space direction followed by gradient spoiling in all three axes. The main difference from the previous implementation [21] is that the gate evaluation (MRI gate) here is performed once with every multi-echo block of NE echoes rather than within a separate evaluation loop that precedes the acquisition of each block of echoes. Note that the MRI gate evaluates the instantaneous level of the respiration control signal such that the MRI TR remains constant and the sequence operates in a true steady-state mode. The hardware limitations of the system dictate that the evaluation must be accompanied by a fixed delay period the order of 1 ms. It should also be noted that the dynamic reacquisition scheme is programmed to reacquire a specific amount of data that were acquired immediately before each breath is first detected by the MRI gate evaluation, and so the evaluation may be positioned before or after the data acquisition statements within the pulse sequence.

**Fig. 1:**
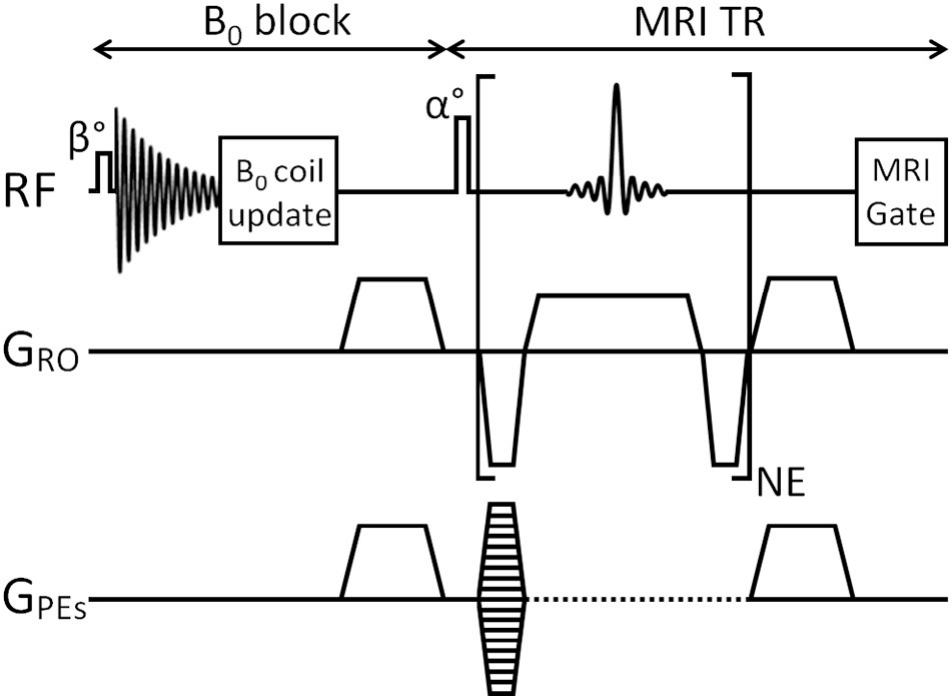
Diagrammatic representation of the MGE 3D scan. An optional *B*_0_ measurement and correction block was inserted before the MGE sequence and an optional respiration gating evaluation period ‘MRI Gate’ was appended to the MGE sequence. NB Phase encoding was only performed once after the MRI excitation pulse.

An optional *B*_0_ measurement block was inserted before each multi-echo block and included a 16 μs hard pulse with FA 1°, a delay of 3 ms during which the NMR signal was routed to a custom built *B*_0_ stabilisation unit, a delay of 0.2 ms during which the *B*_0_ stabilisation unit updated the voltage applied to the *B*_0_ coil, followed by identical gradient spoiling in all three axes, as shown in Fig. 1.

MGE 3D scans with isotopic resolution 176 μm were performed with TR 38 ms, time of 1st echo (TE1) 3 ms, echo spacing (ESP) 3 ms, gradient spoiling of 180 mT/m in all 3 axes for 3 ms, FOV 45 × 22.5 × 22.5 mm^3^ and matrix 256 × 128 × 128. MGE 3D scans with isotopic resolution 117 μm were performed with TR 40 ms, TE1 2.25 ms, ESP 4.25 ms, gradient spoiling of 160 mT/m in all 3 axes for 2.54 ms, FOV 22.5 × 22.5 × 22.5 mm^3^ and matrix 192 × 192 × 192. All phase encoding was applied in a standard linear order.

### 2.2 B_0_ stabilisation

Following the RF excitation at the beginning of each *B*_0_ measurement block, the NMR signal was routed to a custom built *B*_0_ stabilisation unit which mixed the signal to an audio frequency nominally set to 1000 Hz. 1000 Hz was selected as the target frequency to enable an Arduino based single channel receiver system to distinguish between positive and negative frequency shifts, corresponding to warming and cooling, respectively. The user is free to set the target frequency according to the system and application requirements. A dual-supply op-amp within the *B*_0_ stabilisation unit amplified the signal with large gain to resemble a square wave within the bounds of ±5 V. This enabled the frequency shift of the signal to be calculated from the time intervals between the zero crossings in the 3 ms measurement period of the *B*_0_ block, with a digital resolution of 62.5 ns (corresponding to a sampling rate of 16 MHz). Specifically, if the time *t* between the first and last zero crossings is determined (in seconds) for a total of *n* zero crossings then the frequency of the signal is given by (*n* − 1)/2 *t* Hz for *n* ≥ 2. Typically, 5 ≤ *n* ≤ 7 for frequencies close to 1000 Hz. Analogue-to-digital conversion using the Arduino for other types of analysis, e.g. FFT, would limit the digital resolution to about 100 μs.

A fixed number of frequency measurements ranging from 1 to 50 were averaged to improve the accuracy [22] before the data were used to effect correction. Use of a single *B*_0_ measurement for correction would be preferable but proved to be too unstable for robust performance when using the hardware described. The data presented in this report therefore correspond to corrections resulting from the average of 10, 42 or 50 measurements. The average of 42 measurements was selected for MPIO quantification as it was considered to represent an acceptable compromise between the frequency and the accuracy/precision of the update.

The *B*_0_ measurements were respiration gated by discarding any individual shifts ≥6 Hz from the averaging process. The correction (V/Hz) required to adjust measurements back to the target frequency of 1000 Hz was determined by prior calibration of the unit which measured the frequency shift resulting from application of a fixed voltage in a simple pulse and acquire spectroscopy scan.

The correction was added to the standard pre-emphasis subsystem's time dependent *B*_0_ corrections that are applied to the *B*_0_ coil (strength 4.7 × 10^−^^2^ mT/A, inductance 0.03 mH, resistance 0.35 Ω, peak current 5A) in response to all gradient pulse switches. The *B*_0_ coil forms an integral part of all the standard commercially produced gradient sets that were delivered as part of Varian MRI systems. A driver unit delivers the standard *B*_0_ corrections to the *B*_0_ coil according to the differential voltage applied to pins 1 and 2 of a conventional DB9 multi-core shielded cable. Replacement of the supplied single cable with two daisy chained cables provides a convenient break-out connection to the *B*_0_ stabilisation unit where correction according to the measured frequency shift can be applied.

A block diagram showing the components and signal pathways for *B*_0_ stabilisation during scanning on a Varian VNMRS MRI system is provided in Supplementary Fig. 1. The parts of the diagram that belong to the standard Varian MRI system are shaded in grey. Supplementary Fig. 2 provides a circuit diagram of the *B*_0_ stabilisation unit. Supplementary Fig. 3 shows a photo of the assembled components in a 3D printed chassis. The main components of the unit are: MK-3 frequency doubler (Mini-Circuits Europe); SBP-21.4+ bandpass filter (Mini-Circuits Europe); Arduino Uno Rev3, A000066; and RF Mixer Evaluation Board EVAL-AD831EBZ (Analog Devices). Combined with solid state switch ZX80-DR230+ (Mini-Circuits Europe) to switch the NMR signal between the scanner receiver and the *B*_0_ stabilisation unit, and additional sundry electronic components, the total component cost is less than £400.

The detail required to assemble a working *B*_0_ stabilisation unit as described in this report is publically available courtesy of the Bodleian Digital Library Systems and Services of the University of Oxford at https://doi.org/10.5287/bodleian:v0bERQ9G0.

### 2.3 VCAM-MPIO synthesis

Pro-mag™ carboxylic acid MPIO (Cat. No. PMC1N, Lot 10892, Bangs Laboratory Inc., Fishers, IN, USA) were conjugated with azide-free monoclonal rat antibody to mouse VCAM-1 (clone M/K-2, Cat. No. 1510-14, SouthernBiotech, Birmingham, AL, USA) as described previously [3].

### 2.4 In vivo

All animal experiments were approved by the UK Home Office (Animals [Scientific Procedures] Act 1986; PPL 30/3266, 30/3187) and the local Animal Welfare and Ethical Review Body, and conducted in accordance with the University of Oxford Policy on the Use of Animals in Scientific Research and the ARRIVE guidelines [23]. Animals were housed in environmentally enriched and individually ventilated polycarbonate solid-bottomed cages in groups of 5 per cage, in a 12 h day-night cycle facility maintained at 21 ± 2 °C in 55 ± 10% humidity. All mice had ad libitum access to certified food and tap water.

Female, 6–8 week old (14–18 g, *n* = 2) naïve CBA/CaCrl mice (Charles River, UK) were used for technique validation studies. Female, 6–7 week old (15–18 g, *n* = 7) BALB/c mice (Charles River, UK) were used for the mouse neuroinflammation model. BALB/c mice were anesthetized with isoflurane (1–4%) in room air supplemented with oxygen (80%/20% v/v, flow rate 1 L/min), placed in a stereotaxic frame and a burr hole drilled above the injection site (co-ordinates from bregma: anterior 0.5 mm; left 2.5 mm). 1 ng of mouse recombinant interleukin-1β (IL-1β, Peprotech EC, London, UK) in 1 μL sterile PBS was injected into the left striatum, at a depth of 2.5 mm, using a glass microcannula [24]. The scalp incision was sutured and the animals were recovered from anaesthesia. Three hours after surgery, mice were reanaesthetised as above and injected via a tail vein with a dose of 4 mg Fe/kg of VCAM-MPIO in 100 μL sterile PBS. Mice were placed in a custom-built holder and the head immobilised for MRI with a tooth bar and cheek bone pads. Respiration was monitored and maintained at 40–80 breaths/min using a pneumatic balloon (VX010, Viomedex Ltd., UK) positioned against the animal's chest and coupled to a pressure transducer. The respiration signal was passed to a custom-built gating device to generate a threshold based respiration gating control signal to reduce the sensitivity to respiratory motion. Throughout the experiments, mice were maintained at 36 °C core temperature using a resistive heater interfaced to a homeothermic maintenance controller [25]. All animals were killed by a Schedule 1 method at the end of the experiment.

### 2.5 Experimental protocols

Respiration gated MGE 3D scans were performed on a naïve CBA/CaCrl mouse with and without *B*_0_ stabilisation to test the efficacy of *B*_0_ stabilisation. For the scans acquired without *B*_0_ stabilisation the calculated frequency of each *B*_0_ measurement FID was recorded, but feedback to the *B*_0_ coil to effect correction was disabled. Respiration gated MGE 3D scans were performed on a second naïve CBA/CaCrl mouse to assess the effect of averaging the frequency measurements and the effect of respiration gating the *B*_0_ stabilisation frequency measurements.

The effect of respiration gating on the MRI data acquisition was next assessed in the BALB/c neuroinflammation mouse model by performing *B*_0_ stabilised MGE 3D scans with and without respiration gating (*n* = 1). Subsequently, respiration gated MGE 3D scans were performed with and without *B*_0_ stabilisation to detect molecularly targeted MPIO in the BALB/c mouse model of acute neuroinflammation (*n* = 6).

### 2.6 MPIO quantification

Quantification was performed using in-house MATLAB code and manually generated brain masks. Signals arising from ventricles or sinuses, which naturally appear hypointense, were excluded from analyses. The signal-to-noise (SNR) was determined from the signal intensity within the brain after excluding hypointensities (SI_brain_) and the SD of the background noise (SD_noise_) according to SNR = SI_brain_/SD_noise_. The contrast-to-noise (CNR) was determined from an additional measurement of the signal intensity coming from VCAM-MPIO (SI_void_) and calculated according to CNR = (SI_brain_ − SI_void_)/SD_noise_ [3].

## 3 Results and discussion

### 3.1 Efficacy of B_0_ stabilisation

**Fig. 2:**
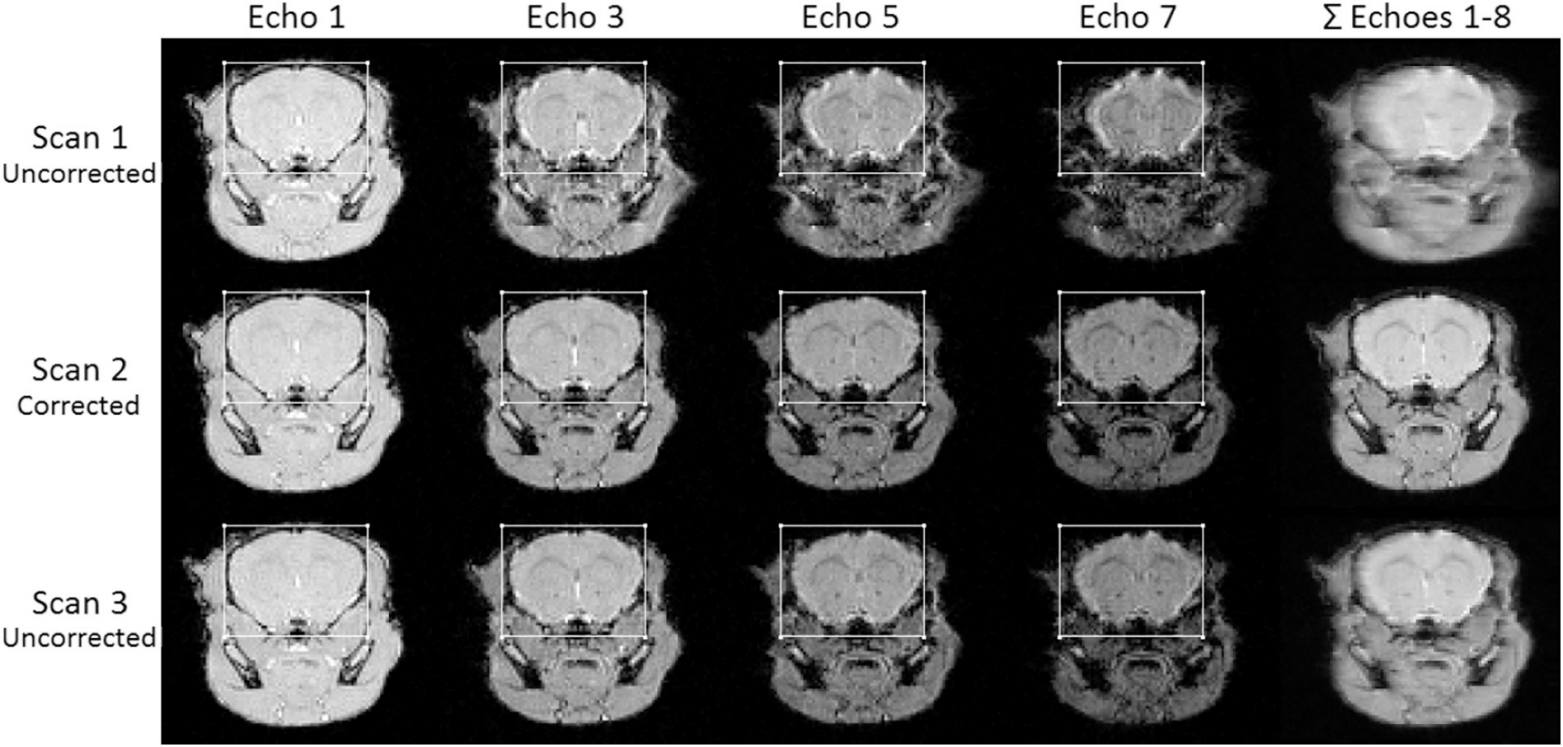
Echoes 1, 3, 5 and 7 from an axial slice taken from three successive respiration gated MGE 3D scans of a naïve mouse acquired with isotopic resolution 176 μm. A composite image generated by simple addition of the magnitude images from the 8 echoes is displayed in the right hand column. A static rectangular region of interest (ROI) is overlaid on the individual echo images. Top row: Scan 1 acquired without *B*_0_ stabilisation in 23.2 min, 315 Hz *B*_0_ drift. Middle row: Scan 2 acquired with *B*_0_ stabilisation in 23.2 min, 120 Hz *B*_0_ drift. Bottom row: Scan 3 acquired without *B*_0_ stabilisation in 20.9 min, 100 Hz *B*_0_ drift. The fast phase encode dimension runs vertically and the slow phase encode dimension runs horizontally. Later echo images are progressively shifted in the slow phase encode dimension and distorted in the absence of *B*_0_ stabilisation.

Fig. 2 shows the odd echoes from an axial slice taken from three successive respiration gated MGE 3D scans of a naïve CBA/CaCrl mouse acquired with inclusion of the *B*_0_ measurement block at an isotopic resolution of 176 μm.

The 315 Hz *B*_0_ drift during scan 1 severely degrades image alignment of the echoes in the slow phase encoding dimension with respect to the first echo. An increasing mixture of translation, scaling and blurring is evident with increasing TE which compromises the composite image and limits the image resolution that is realised. The 120 Hz *B*_0_ drift over the second scan is mitigated by the correction and the echoes are properly aligned. The small apparent reduction in the size of the brain in the corrected Scan 2 image of Echo 7 (with respect to Echo 1) is due to a progressive signal loss at the surface of the brain caused by the local change in magnetic susceptibility and is compounded by the image display of echoes with uniform brightness and contrast. Without the correction the distortions in scan 2 would be at least as severe as in scan 3 which experienced a 100 Hz *B*_0_ drift and resulted in misalignment of later echoes by approximately two pixels. The duration of scan 3 was slightly less than that of the previous scans due to a spontaneous reduction in the free breathing respiration rate. The *B*_0_ drift experienced during scan 1 was much greater than experienced during subsequent scans because scan 1 followed a lengthy period of scanner inactivity.

### 3.2 Frequency measurement

**Fig. 3:**
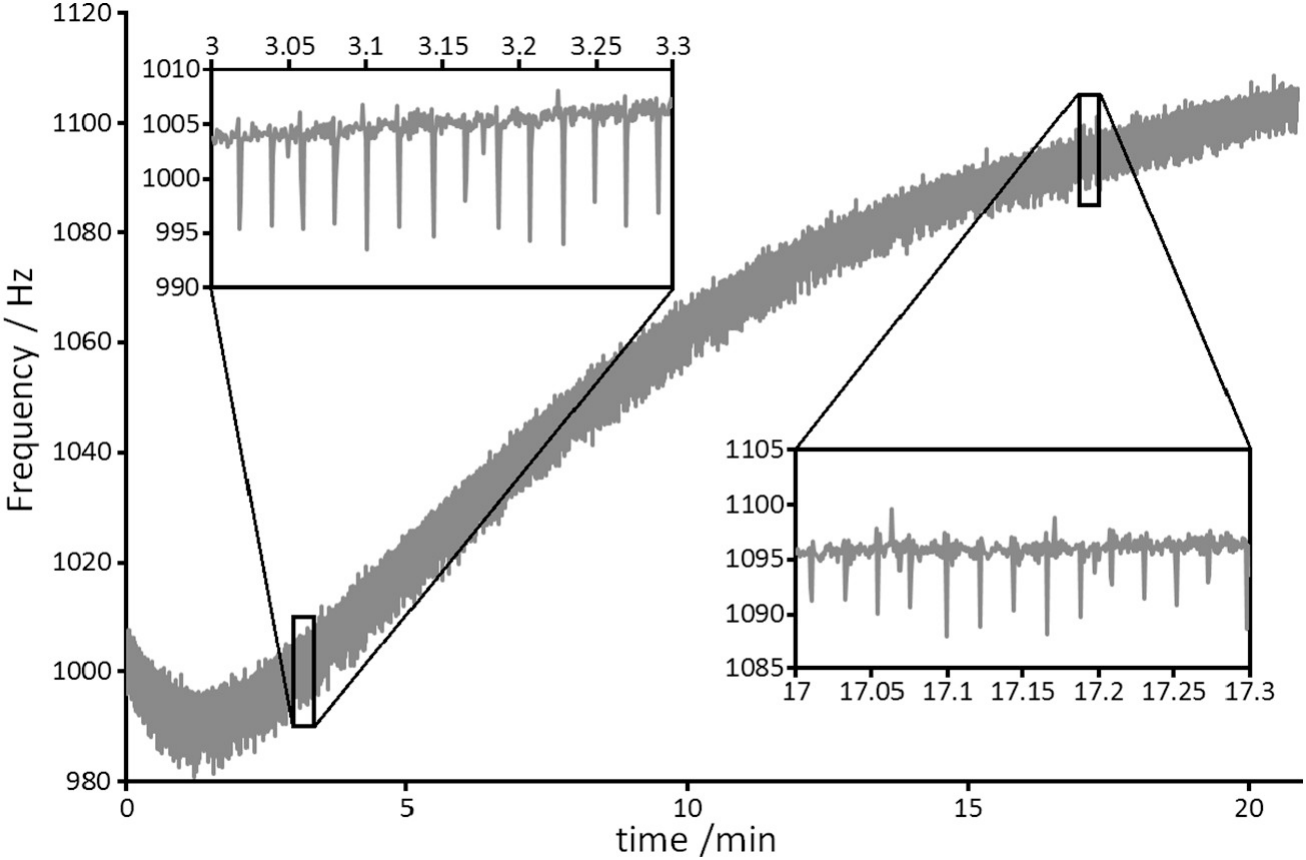
Frequency measurements during scan 3 from three successive respiration gated MGE 3D scans of a naïve mouse. The overall frequency drift experienced was about 100 Hz due to the temperature dependence of the *B*_0_ field. The short time constant cyclic frequency drifts evident in the insert plots are induced by respiration. The full plot for the 20.9 min scan corresponds to 28,338 data points in total.

The recorded frequency measurements during scan 3 are plotted in Fig. 3. The initial negative frequency drift was caused by cooling during a 3 min hiatus in scanning whilst the data of scan 2 were reconstructed, briefly inspected, and stored. After about a minute of scanning the effect of system heating once again becomes dominant. The extent and nature of the overall drift depends on the magnet hardware, scan duty cycle, and prior use of the scanner. As such, it would be difficult to predict the change in frequency during a scan in order to correct data retrospectively, although that would be possible if the frequency data are recorded. Standard image registration methods using the first echo as target are unlikely to be able to produce satisfactory image correction given the nature of the artefacts that are evident in later echoes. The direct correction presented here follows the guiding principle that the best way to remove artefacts is to avoid them altogether [26]. Furthermore, it should be noted that frequency offsets for RF pulse transmission in slice and chemical shift selections cannot be corrected retrospectively and require that the field and/or frequency offsets be stabilised in real time [20].

Close inspection of the fine detail in the curve shows that the frequency measurement is modulated by the animal's respiration, as highlighted by the insert plots of Fig. 3. In the *B*_0_ corrected scan adjustment was made to the *B*_0_ coil after averaging 42 fresh frequency measurements whilst rejecting measurements that differed by ≥6 Hz from the target frequency in order to minimise the adverse effect of respiration. Averaging the measurements serves to improve the accuracy with which the correction is made by reducing the jitter in the correction. A random frequency jitter > 1 Hz in a low duty cycle phantom scan was observed to produce a mottled image and ghosting in the later echoes (data not shown). The averaging of 42 measurements approximately every 2 s was selected to be large enough to reduce the jitter, yet small enough to avoid large correction steps when the frequency change is most severe. The fastest rate of frequency change recorded was <0.5 Hz/s, and so further improvement to the time resolution of the correction, using for example a moving average, was not deemed necessary.

The direct effect of respiration on the NMR signal itself is considered to provide the best opportunity for identifying signal corruption due to respiration in real time. Physiological measurement with a pressure balloon typically results in a time difference between the effect on the NMR signal from the tissue of interest and the detection of respiration that depends upon the positioning of the device, and also incurs delays from pneumatic transmission and the application of low pass audio filters which are typically the order of 50 ms.

**Fig. 4:**
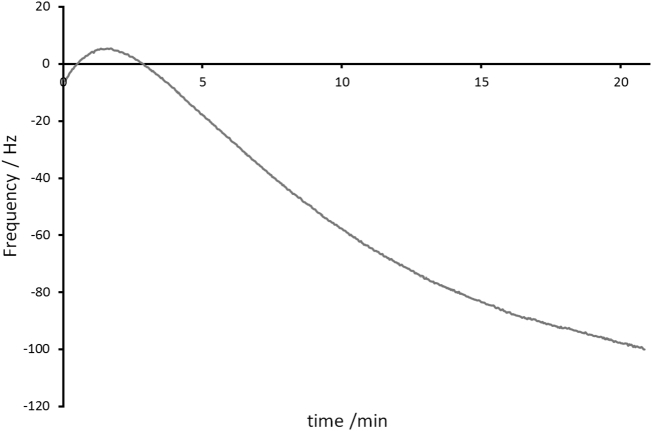
The frequency correction that would be applied by the *B*_0_ stabilisation unit when presented with the frequency measurements presented in Fig. 3 when averaging 42 fresh measurements and discarding any individual shifts ≥ 6 Hz from the previous average. The calibrated correction of 0.513 mV/Hz is what would be added to the voltage that is applied to the *B*_0_ coil. The plot consists of 642 data points in total, which would correspond to an adjustment being made approximately every 2 s.

Fig. 4 shows the correction that the *B*_0_ stabilisation unit would apply given the *B*_0_ measurement data presented in Fig. 3.

### 3.3 Respiration gated B_0_ stabilisation

**Fig. 5:**
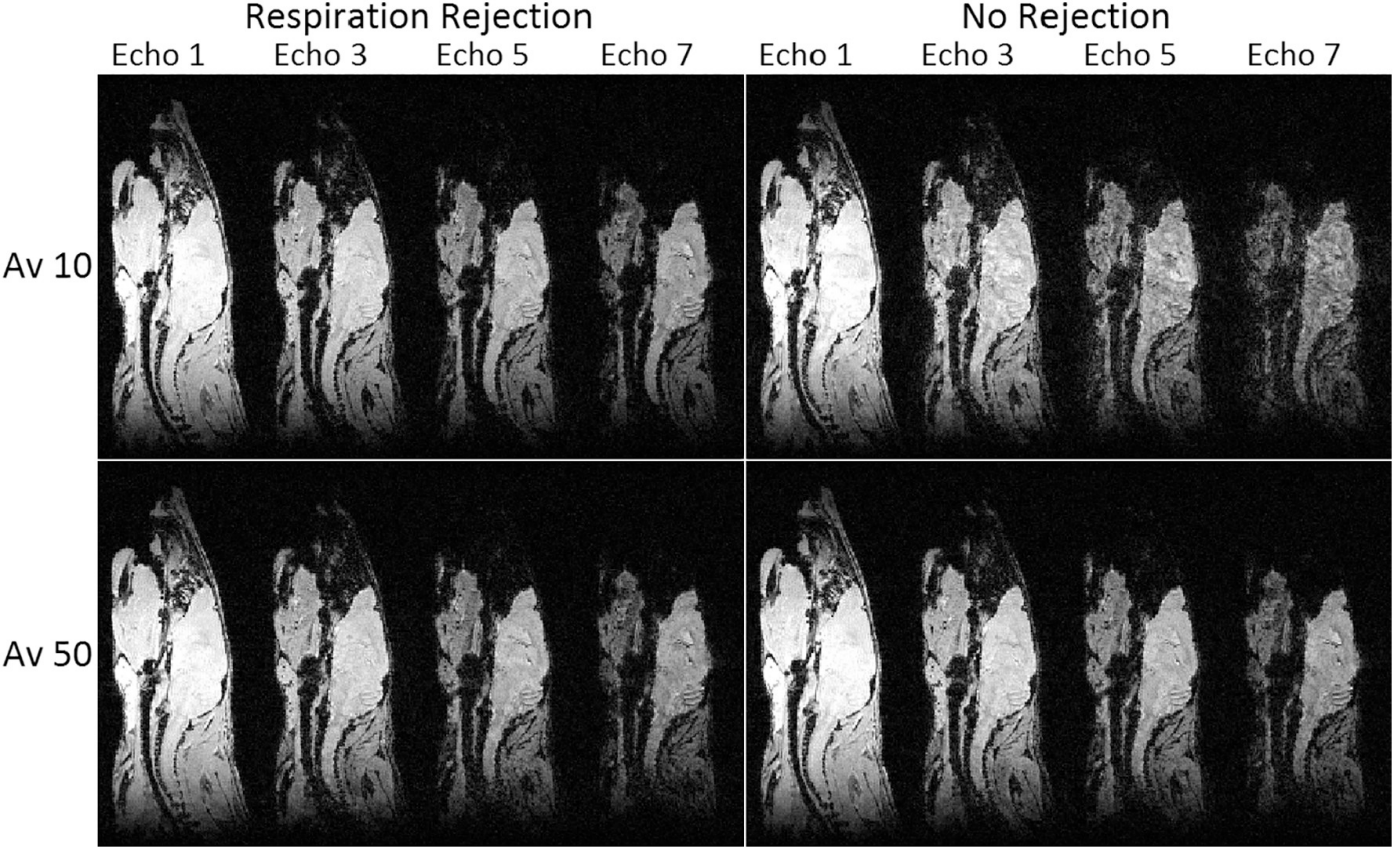
Sagittal slices taken from MGE 3D scans of a naïve mouse acquired with isotopic resolution 176 μm. Each panel shows (from left to right) echoes 1, 3, 5 and 7 from a central sagittal slice. *B*_0_ stabilisation was performed as follows. Left panels: rejection of measurements ≥ 6 Hz from the previous average. Right panels: no rejection of measurements. Top panels: averaging 10 fresh frequency measurements. Bottom panels: averaging 50 fresh frequency measurements. Respiratory gating was applied to the image data acquisition regardless of whether or not it was used for the *B*_0_ corrections.

The effect of rejecting measurements that differed by ≥6 Hz from the previous average in order to minimise the adverse effect of respiration is demonstrated in Fig. 5. The left hand panels show *B*_0_ stabilisation performed with measurement rejection and averaging: 10 measurements (top panel); 50 measurements (bottom panel). The right hand panels show *B*_0_ stabilisation performed without measurement rejection and averaging: 10 measurements (top panel); 50 measurements (bottom panel).

When *B*_0_ stabilisation is performed by averaging 10 fresh frequency measurements the rejection of measurements ≥ 6 Hz from the previous average is able to eliminate measurements that are corrupted by respiration. Without rejection the unstable average results in a mottled image and ghosting. When *B*_0_ stabilisation is performed by averaging 50 fresh frequency measurements the effect of including measurements during respiration is diluted to become virtually negligible. The fidelity of the spinal cord in the later echoes provides a good visual comparison of image quality. The averaging of 10 fresh frequency measurements is able to provide the best stability but, in our current implementation, it is not sufficiently robust. This lack of robustness is because the initial correction can be dominated by measurements that are corrupted by respiration, with the result that subsequent measurements based on stable inter-breath periods are rejected. The drawback of prolonged averaging is that instabilities can be introduced if the correction steps are large. On the system used in this work, averaging of 42 measurements enabled a robust level of performance whilst keeping correction steps sufficiently small.

### 3.4 Respiration gated MRI

**Fig. 6:**
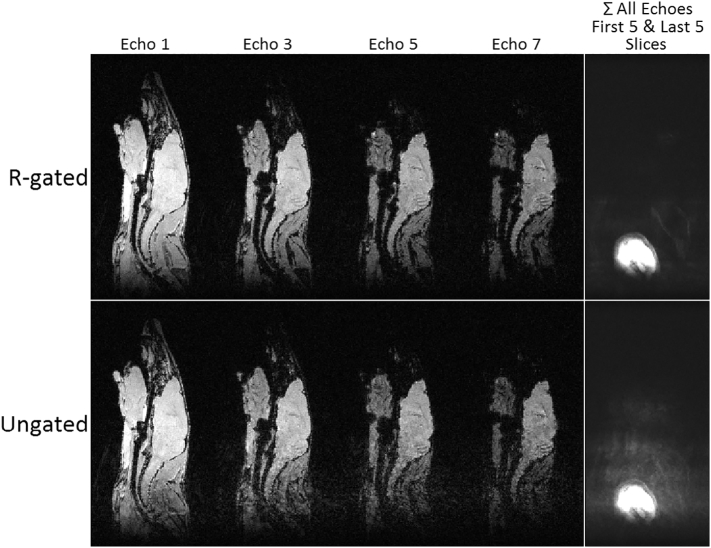
Sagittal slices from MGE 3D scans of a BALB/c neuroinflammation mouse model acquired with isotopic resolution 176 μm and *B*_0_ stabilisation. Top row: respiration gated, scan time 21.1 min. Bottom row: ungated, scan time 12.2 min. Left panels: echoes 1, 3, 5 and 7 from the central sagittal slice. Right hand column: sum of all echoes from first five and last five sagittal slices. The brightest region is part of the neck. In the ungated scan the surrounding signal is largely respiration induced ghosting.

Fig. 6 shows a comparison between two MGE 3D scans acquired from the BALB/c neuroinflammation mouse model acquired with *B*_0_ stabilisation. The top row shows results from a 21.1 min scan with respiration gating and reacquisition and the bottom row shows results from a 12.2 min ungated scan. Although a respiration rate of about 60 breaths per minute results in a gated scan time that is almost double that of the ungated scan [21], good image fidelity is preserved to provide high resolution detail, especially towards the back of the brain. The summed sagittal slices in the right hand column show a significant level of ghosted image intensity in the case of ungated scanning, which is unavoidable, because the head restraint fixes the skull in position but other organs and structures are free to move during respiration. In essence, although the skull is securely fixed in place, the fidelity of brain imaging is improved with respiration gating, even for scan modes that are traditionally considered robust to motion.

### 3.5 Detection of iron oxide particles

**Fig. 7:**
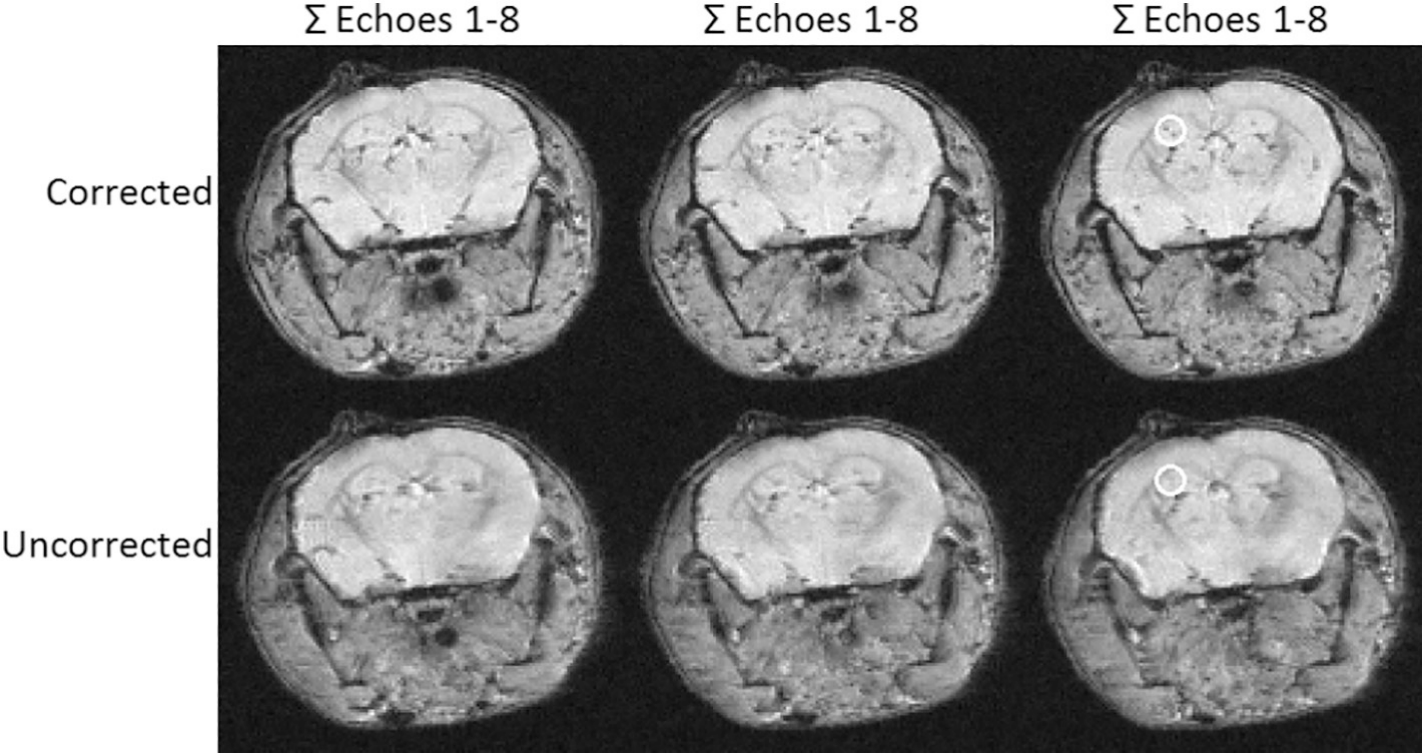
Example high resolution axial slices generated with echo combination by simple addition from respiration gated MGE 3D scans of targeted MPIO in a neuroinflammation mouse model acquired with isotopic resolution 117 μm. Top row: *B*_0_ stabilisation of a 345 Hz drift, scan time 39.8 min. Bottom row: uncorrected 320 Hz drift, scan time 36.6 min. The circled region in the third slice highlights two clusters of MPIO in the corrected scan that are not evident in the uncorrected scan.

Following validation of the technique in naïve mice, the methods were applied to detect VCAM-MPIO in the neuroinflammation mouse model. Echo combination was used for the purpose of MPIO quantification since it has previously been shown to be more effective than quantification using data from a single echo [3]. Fig. 7 compares high resolution composite axial slices generated by simple echo addition from respiration gated MGE 3D scans acquired with and without *B*_0_ stabilisation and subject to 345 Hz and 320 Hz drifts, respectively. The composite image resolution approaches the prescribed resolution with *B*_0_ stabilisation enabling good detection and visualisation of the targeted MPIO. Without *B*_0_ stabilisation the composite image resolution is compromised through image misalignment and blurring such that only larger clusters of MPIO can be faithfully detected. Quantification of the composite images resulted in a 4% increase in SNR, a 5% increase in contrast-to-noise ratio (CNR), and a 48% increase in the total number of hypointense image voxels assigned to MPIO, when data were acquired with *B*_0_ stabilisation.

Although the *B*_0_ stabilisation scheme used here incurred a 14% time penalty to accommodate the additional *B*_0_ measurement block, the overall improvement in image fidelity more than compensates for the scan time overhead. Changes in the free breathing respiration rate during scanning result in variation about this nominal value. Pre-warming the 9.4 T system used to obtain the results presented here is not a practical option because, as the results of Fig. 1 demonstrate, the *B*_0_ drift experienced after virtually continuous scanning for 45 min still compromises the composite image. The respiration gated image acquisition typically imposes a 50–100% scan time overhead.

The full respiration gated MGE 3D data sets for six mice acquired with and without *B*_0_ stabilisation for this report are publically available courtesy of the Bodleian Digital Library Systems and Services of the University of Oxford at https://doi.org/10.5287/bodleian:bpkMrMOak. The data are in NIfTI-1.1 format (http://nifti.nimh.nih.gov/) and can be viewed with ImageJ (https://imagej.nih.gov/ij/). It is particularly instructive to inspect the registration of image data by selecting a slice and scrolling through the acquired echoes. It can readily be seen that the data comparison presented in Fig. 5 is representative.

The *B*_0_ stabilisation method and hardware used here were selected due to the relative ease of implementation and maintained the *B*_0_ field with an accuracy of about 1 Hz. Other stabilisation methods may enable a superior level of performance. In particular, an independent mechanism, akin to the deuterium lock used in high resolution NMR spectroscopy, ought to permit accurate correction in all pulse sequences with negligible scan time overhead. Indeed, the optimum solution is considered to be a fully integrated correction within the commercial hardware provided by system manufacturers. In instances where the system does not include a *B*_0_ coil this can be achieved by suitable adjustment of the internal reference frequency. To a first approximation, the performance of *B*_0_ stabilisation and internal reference frequency adjustment are equivalent. However, *B*_0_ stabilisation requires the availability of a *B*_0_ coil within the system, whilst internal reference frequency adjustment potentially renders the system susceptible to frequency dependent effects, such as RF coil performance. Nevertheless, in the first instance, there is no doubt that deployment of either method is able to significantly improve system stability. In essence, the work presented here serves to highlight the requirement to effect good *B*_0_ stabilisation throughout MGE 3D scanning to enable improved detection and visualisation of molecularly targeted iron oxide particles. The hardware developed for this work could, of course, be applied to all scan modes in all applications, but would likely require case-specific sequence modifications to realise a suitable *B*_0_ stabilisation method.

## 4 Conclusion

The methods presented minimise the image artefacts that arise from a gradient demand derived *B*_0_ instability and respiratory motion. In particular, high resolution imaging for the detection of molecularly targeted iron oxide particles in the mouse brain requires good stabilisation of the main *B*_0_ field, and can benefit from a respiration gated image acquisition strategy. It is anticipated that *B*_0_ field stabilisation will improve imaging studies using molecularly targeted MPIO as it enables the resulting MGE 3D composite image resolution to approach the prescribed image resolution.

## CRediT authorship contribution statement

**Stuart Gilchrist:**Conceptualization, Methodology, Software, Validation, Writing - original draft.**Paul Kinchesh:**Conceptualization, Methodology, Software, Validation, Writing - original draft.**Niloufar Zarghami:**Investigation, Writing - review & editing.**Alexandre A. Khrapitchev:**Investigation, Writing - review & editing.**Nicola R. Sibson:**Supervision, Writing - review & editing.**Veerle Kersemans:**Investigation, Writing - review & editing.**Sean C. Smart:**Conceptualization, Methodology, Supervision, Writing - original draft.

## Appendix A Supplementary data

Supplementary figures Image 1

## References

[1] McAteer M.A., Sibson N.R., von Zur Muhlen C., Schneider J.E., Lowe A.S., Warrick N. (2007). In vivo magnetic resonance imaging of acute brain inflammation using microparticles of iron oxide. Nat Med.

[2] Perez-Balderas F., van Kasteren S.I., Aljabali A.A., Wals K., Serres S., Jefferson A. (2017). Covalent assembly of nanoparticles as a peptidase-degradable platform for molecular MRI. Nat Commun.

[3] Zarghami N., Khrapitchev A.A., Perez-Balderas F., Soto M.S., Larkin J.R., Bau L. (2018). Optimization of molecularly targeted MRI in the brain: empirical comparison of sequences and particles. Int J Nanomedicine.

[4] Van Kasteren S.I., Campbell S.J., Serres S., Anthony D.C., Sibson N.R., Davis B.G. (2009). Glyconanoparticles allow pre-symptomatic in vivo imaging of brain disease. Proc Natl Acad Sci.

[5] Hoyte L.C., Brooks K.J., Nagel S., Akhtar A., Chen R., Mardiguian S. (2010). Molecular magnetic resonance imaging of acute vascular cell adhesion molecule-1 expression in a mouse model of cerebral ischemia. J Cereb Blood Flow Metab.

[6] Serres S., Mardiguian S., Campbell S.J., McAteer M.A., Akhtar A., Krapitchev A. (2011). VCAM-1-targeted magnetic resonance imaging reveals subclinical disease in a mouse model of multiple sclerosis. FASEB J.

[7] Serres S., Soto M.S., Hamilton A., McAteer M.A., Carbonell W.S., Robson M.D. (2012). Molecular MRI enables early and sensitive detection of brain metastases. Proc Natl Acad Sci.

[8] McAteer M.A., Mankia K., Ruparelia N., Jefferson A., Nugent H.B., Stork L.-A. (2012). A leukocyte-mimetic MRI contrast agent homes rapidly to activated endothelium and tracks with atherosclerotic lesion macrophage content. Arterioscler Thromb Vasc Biol.

[9] Cheng V.W.T., Soto M.S., Khrapitchev A.A., Perez-Balderas F., Zakaria R., Jenkinson M.D. (2019). VCAM-1 targeted magnetic resonance imaging enables detection of brain micrometastases from different primary tumours. Clin Cancer Res.

[10] Lauterbur P.C., Bernardo M.L., Mendonca Dias M.H., Hedges L.K. (1986). Microscopic NMR imaging of the magnetic fields around magnetite particles. Proc Intl Soc Mag Reson Med.

[11] Ward J., Guthrie J.A., Wilson D., Arnold P., Lodge J.P., Toogood G.J. (2003). Colorectal hepatic metastases: detection with SPIO-enhanced breath-hold MR imaging - comparison of optimized sequences. Radiology.

[12] Choi J.S., Kim M.J., Kim J.H., Choi J.Y., Chung Y.E., Park M.S. (2010). Comparison of multi-echo and single-echo gradient-recalled echo sequences for SPIO-enhanced liver MRI at 3 T. Clin Radiol.

[13] Schieda N., Avruch L., Shabana W.M., Malone S.C. (2015). Multi-echo gradient recalled echo imaging of the pelvis for improved depiction of brachytherapy seeds and fiducial markers facilitating radiotherapy planning and treatment of prostatic carcinoma. J Magn Reson Imaging.

[14] Kinchesh P., Gilchrist S., Zarghami N., Khrapitchev A.A., Sibson N.R., Smart S.C. (2018). Respiratory-gated *B*_0_ field stabilisation for high resolution mouse brain imaging. Proc Intl Soc Mag Reson Med.

[15] Hoult D.I., Richards R.E., Styles P. (1978). A novel field-frequency lock for a superconducting spectrometer. J Magn Reson.

[16] Weavers P.T., Tao S., Trzasko J.D., Frigo L.M., Shu Y., Frick M.A. (2018). B_0_ concomitant field compensation for MRI systems employing asymmetric transverse gradient coils. Magn Reson Med.

[17] Vos S.B., Tax C.M., Luijten P.R., Ourselin S., Leemans A., Froeling M. (2017). The importance of correcting for signal drift in diffusion MRI. Magn Reson Med.

[18] Alhamud A., Taylor P.A., van der Kouwe A.J., Meintjes E.M. (2016). Real-time measurement and correction of both B0 changes and subject motion in diffusion tensor imaging using a double volumetric navigated (DvNav) sequence. Neuroimage.

[19] Price A.N., Malik S.J., Broadhouse K.M., Finnemore A.E., Durighel G., Cox D.J. (2013). Neonatal cardiac MRI using prolonged balanced SSFP imaging at 3T with active frequency stabilization. Magn Reson Med.

[20] Benner T., van der Kouwe A.J., Kirsch J.E., Sorensen A.G. (2006). Real-time RF pulse adjustment for *B*_0_ drift correction. Magn Reson Med.

[21] Kinchesh P., Gilchrist S., Beech J.S., Gomes A.L., Kersemans V., Newman R.G. (2018). Prospective gating control for highly efficient cardio-respiratory synchronised short and constant TR MRI in the mouse. Magn Reson Imaging.

[22] Lee J., Santos J.M., Conolly S.M., Miller K.L., Hargreaves B.A., Pauly J.M. (2006). Respiration-induced B0 field fluctuation compensation in balanced SSFP: real-time approach for transition-band SSFP fMRI. Magn Reson Med.

[23] Kilkenny C., Browne W.J., Cuthill I.C., Emerson M., Altman D.G. (2010). Improving bioscience research reporting: the ARRIVE guidelines for reporting animal research. PLoS Biol.

[24] McAteer M.A., Schneider J.E., Ali Z.A., Warrick N., Bursill C.A., von zur Muhlen C. (2008). Magnetic resonance imaging of endothelial adhesion molecules in mouse atherosclerosis using dual-targeted microparticles of iron oxide. Arterioscler Thromb Vasc Biol.

[25] Gilchrist S., Gomes A.L., Kinchesh P., Kersemans V., Allen P.D., Smart S.C. (2016). An MRI-compatible high frequency AC resistive heating system for homeothermic maintenance in small animals. PLoS One.

[26] Smith T.B., Nayak K.S. (2010). MRI artifacts and correction strategies. Imaging Med.

