# Supplementary material for: Improved detection of molecularly targeted iron oxide particles in mouse brain using B0 field stabilised high resolution MRI
Source: Magn Reson Imaging. 2020 Apr;67:101–8. doi: 10.1016/j.mri.2020.01.002 (PMC7049896; doi:10.1016/j.mri.2020.01.002)
Supplement: Supplementary file 1 — Supplementary figures [file mmc1.docx]

*Supplementary*


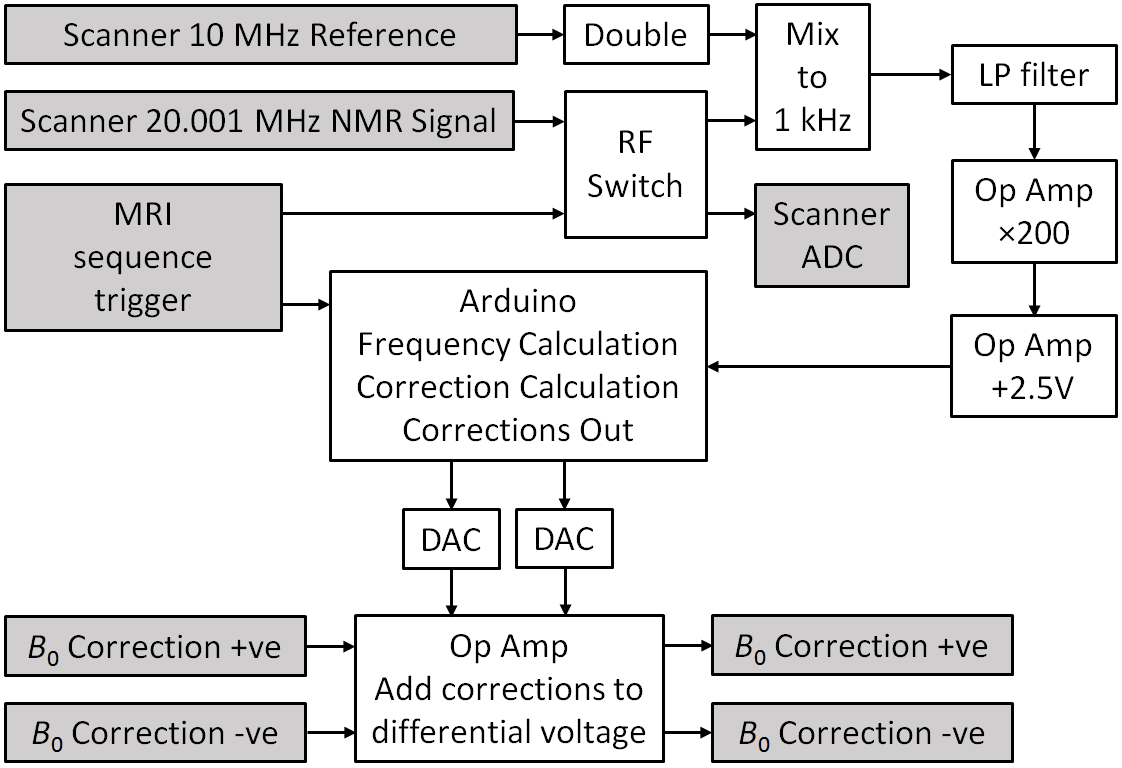


**Supplementary** **Fig. 1**. Block diagram showing the components and signal pathways for *B*_0_ stabilisation during scanning on a Varian VNMRS MRI system. The shaded portions correspond to components that form part of the standard Varian VNMRS MRI system.

**
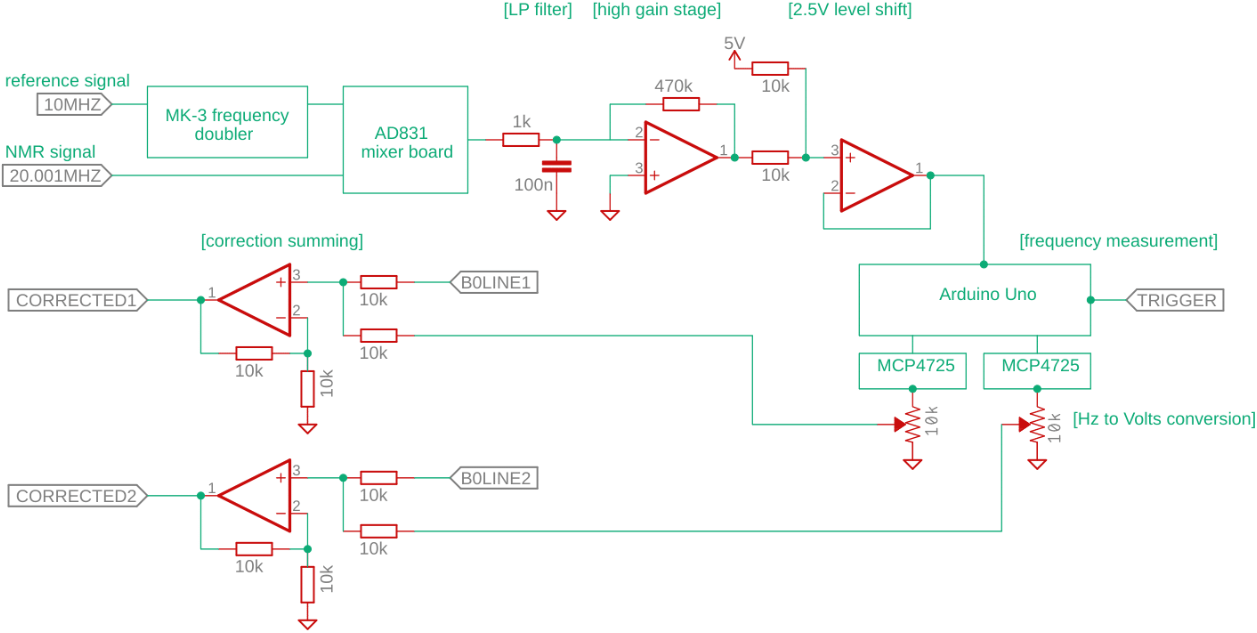
**

**Supplementary** **Fig. 2**. Circuit diagram of the *B*_0_ stabilization unit.

**
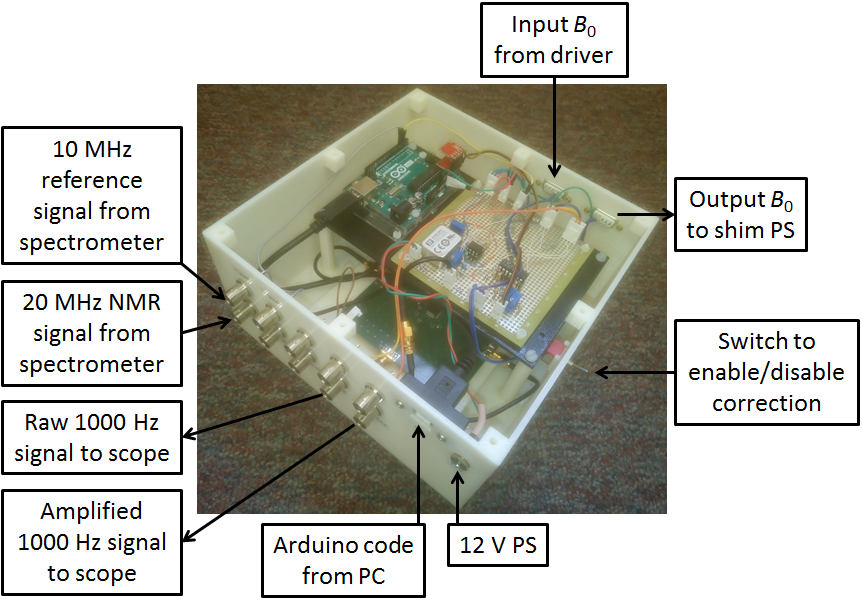
**

**Supplementary** **Fig. 3**. Assembled components of the *B*_0_ stabilisation unit inside a 3D printed chassis.
